# THE ROLE OF FORMAL RESOURCES ON ELDER MISTREATMENT HELP-SEEKING BEHAVIORS AMONG ASIAN OLDER ADULTS

**DOI:** 10.1093/geroni/igac059.2293

**Published:** 2022-12-20

**Authors:** Eun Jeong Lee, Ga-Young Choi, Eun Koh

**Affiliations:** Asian American Resource and Information Network, Wood Ridge, New Jersey, United States; California State University, Los Angeles, Los Angeles, California, United States; The Catholic University of America, Washington, District of Columbia, United States

## Abstract

Elder mistreatment is one of the most difficult topics to talk about among Asian older adult immigrants in the U.S. because they are unfamiliar and uncomfortable to disclose the issue due to their unique cultural values and limited English language proficiency. We recruited 494 community dwelling older adults from five cities who identified themselves of Asian ethnicity, and were 55 years old and older. Participants had various Asian ethnic and language backgrounds (e.g. Korean, Chinese, Filipino, and Vietnamese). Most of them were foreign-born (84.6%) with the mean age of 69.0 (SD = 8.47). Using the participant survey data, we focused on examining difference regarding elder mistreatment experience, perceptions, and help-seeking intentions between the Senior Community Service Employment Program (SCSEP) group and the non-SCSEP group that may or may not participate in paid workforce. The SCSEP is the federally paid workforce program for low-income older adults aged 55 or above aiming at fostering older adults’ community service and economic self-sufficiency. There was a significant difference in emotional mistreatment experiences between SCSEP and non-SCSEP participants. Furthermore, the two groups were significantly different in their likelihood of seeking help from Adult Protective Services (APS). Those who ever participated in the SCSEP were more likely to seek help from APS (X2 = 1, N = 284) = 4.674, p ≤ .05. Implications for future research and gerontology services are discussed to enhance Asian older adults’ awareness of elder mistreatment, and APS and other formal sources of help.

